# Correlation of the oxygen radical activity and antioxidants and severity in critically ill surgical patients – study protocol

**DOI:** 10.1186/1749-7922-8-18

**Published:** 2013-05-03

**Authors:** Hongjin Shim, Ji Young Jang, Seung Hwan Lee, Jae Gil Lee

**Affiliations:** 1 Department of Surgery, Yonsei University Wonju College of Medicine, Wonju, Korea; 2 Department of Surgery, Yonsei University College of Medicine, 50 Yonsei-ro, Seodaemun-gu, Seoul, 120-752, Korea

**Keywords:** Oxygen radical activity, Antioxidant activity, Sepsis, Critically ill patient, Zinc, Selenium, Glutamate

## Abstract

**Background:**

Surgical patients who require an emergent operation commonly have severe sepsis or septic shock, followed by high morbidity and mortality rates.

Despite advances in treatment however, no predictable markers are available. In severe sepsis, many pathophysiologic mechanisms are involved in progression to organ failure, and oxygen free radical and antioxidants are known to contribute to this process. Oxygen free radical and antioxidants contribute to progression of organ failure in severe sepsis. In fact, oxygen radical activity has been reported to be correlated with disease severity and prognosis in patients with severe sepsis or septic shock. Accordingly, we aim to assess the usefulness of oxygen free radical and antioxidant concentrations to predict the disease severity and mortality in a cohort of critically ill surgical patients.

**Methods/Design:**

This is a prospective observation study including patient demographic characteristics, clinical information, blood sampling/serum oxygen radical activity, serum antioxidant activity, serum antioxidant concentrations (zinc, selenium and glutamate), disease severity scores, outcomes, lengths of stay in intensive care unit, hospital 30-day mortality.

## Background

Critically ill surgical patients usually have a septic status combined with severe systemic inflammation and shock. Sepsis is commonly caused by a gastrointestinal tract perforation, bowel ischemia, or postoperative complications, such as, pneumonia, intra-abdominal infection, or anastomotic leakage. Severe systemic inflammation and sepsis can cause organ failure with high risk of mortality (4 ~ 15% vs. 1%). Sepsis affects about 18 million people annually, with a mortality rate of 25% for uncomplicated cases and 80% in patients developing multi-organ failure syndrome [1].

Prognostic markers like natriuretic peptide (NP), B-type natriuretic peptide (BNP), or pro-BNP are used to predict postoperative cardiac complications after cardiac or non-cardiac surgery, while procalcitonin is commonly used as prognostic marker and indicator of mortality and antibiotics usage in septic patients. In addition, lactate clearance was recently reported to be a useful indicator of resuscitation and prognosis in severe sepsis [2,3]. Furthermore, some scoring systems, such as, the acute physiologic and chronic health evaluation (APACHE) II, the sequential organ failure assessment (SOFA), and multiple organ dysfunction score (MODS) systems, are also used to evaluate critically ill patient’s condition. However, no clinically adaptable markers, except lactate clearance and procalcitonin, are available for determining the outcomes of critically ill surgical patients with severe sepsis. Inflammatory processes after infection are known to involve cells, inflammatory mediators, cytokines, pro-inflammatory substances, nitric oxide, arachidonic acid metabolites, and oxygen free radicals. These mediate and induce organ injury leading to organ failure [4-10]. Recently, many reports have been issued on the roles of oxygen free radicals and antioxidants, such as, glutamine, zinc, and selenium, which act as cofactors of glutathione peroxidase [11,12]. Oxygen free radicals (OFR) cause oxidative damage in cells, which lead to DNA damage and mitochondrial dysfunction culminates in cell death [13-15]. There is evidence that oxidative stress caused by reactive oxygen species(ROS) in sepsis is characterized by tissue ischemia reperfusion injury and intense systemic inflammatory response [16-19]. Furthermore, oxidative stress and OFR impair the microcirculation, which induce acute renal failure, and have been correlated with sepsis severity and sepsis-induced morbidity. In sepsis, the protective role of antioxidants against oxidative stress has been known for more than 15 years [20-22]. Supplementation with antioxidants, such as, glutamine, zinc, and selenium may decrease oxidative stress and increase antioxidant activity, but apparently, do not affect mortality [23-28]. Early recognition of oxidative damage in sepsis by assessment of oxidative stress biomarkers is an actual topic for future research [29,30].

## Methods

### Aim

The purpose of the study is to assess the usefulness of the concentration of the oxygen free radical and antioxidants to predict the severity and mortality of the critically ill surgical patients.

### Study population

This prospective study will be performed over 2-year periods (May 2012 ~ April 2014) in single institution. About 50 patients having severe sepsis or septic shock requiring emergency operation due to the bowel perforation or strangulation will be included.

### Study design

This study will not change or modify the clinical practices of the participitating physicians. Patients are enrolled after acquisition of the informed consent approved by a Severance hospital institutional review board (Approval No. of IRB: 4-2012-0188).

Blood sample is drawn at 1st day, 3rd day, and 7th day after admitting to intensive care unit (ICU) regardless of the disposition of the patients after discharge from the ICU.

The primary endpoint of this study is to evaluate the correlation of the level of oxygen radical activity and severity of the patients. And secondary endpoints are (1) correlation of the level of oxygen radical activity and outcome, i.e., LOS in ICU and hospital, 30 day mortality, in-hospital mortality; (2) correlation of the level of antioxidant and severity and outcome of the patients; (3) relationship of the level of the oxygen radical activity and antioxidants.

### Data collection

Investigators have collected the data including the followings: (1) patient characteristics, i.e., demographic data, severity of sepsis (severe sepsis or septic shock), presence of shock; (2) severity score for 7 days in ICU, i.e., APACHE II score, SOFA score, MODS; (3) clinical progress, i.e., vital signs, daily intake and output; (4) clinical outcomes, i.e., duration of shock, use of mechanical ventilation (MV), duration of MV, length of stay(LOS) in ICU, LOS in hospital, 30 day mortality, in-hospital mortality, complications. Blood samples are drawn to check the level of oxygen radical activity, antioxidation activity, level of the antioxidant (zinc, selenium, and glutamate) (Table 1).

**Table 1:** Collection of dataset of the enrolled patients

Day of ICU^* ^**admission**	APACHE II^** ^**score**	**Severity scoring (MODS**^**†**^**, SOFA**^**‡**^**)**	**Clinical courses (Vasopressors, Shock, MV**^**§**^**, Complications)**	**Oxygen radical activity and antioxidation activity**	**Antioxidants (Zn**^**∥**^**, Se**^**¶**^**, Glutamate)**
1st day	O	O	O	O	O
2nd day			O		
3rd day		O	O	O	O
4th day			O		
5th day			O		
6th day			O		
7th day		O	O	O	O

^*^ICU intensive care unit, ^**^APACHE II acute physiology and chronic health evaluation II, ^†^*MODS* multi-organ dysfunction score, ^‡^*SOFA* sequential organ failure assessment, ^§^*MV* mechanical ventilation, ^∥^*Zn* zinc, ^¶^*Se* selenium.

Oxygen radical activity and antioxidation activity are assessed using CR3000® (Callegari 1930, Italy). Free oxygen radicals test (FORT) kit check the serum H2O2 level directly as oxygen radical. Free oxygen radicals detection (FORD) kit assess the antioxidation activity that check the reactivity with vitamic C, Trolox, albumin, and glutathione to free radical – chromogen.

The levels of the zinc, selenium and glutamate are assessed in the laboratory.

### Statistical analysis

The results will be expressed as standard statistical metrics: median (range), mean ± standard deviation for continuous variables.

The primary endpoint of this study is to evaluate the correlation of the level of oxygen radical activity and severity of the patients.

And secondary endpoints are (1) correlation of the level of oxygen radical activity and outcome, i.e., LOS in ICU and hospital, 30 day mortality, in-hospital mortality; (2) correlation of the level of antioxidant and severity and outcome of the patients; (3) relationship of the level of the oxygen radical activity and antioxidants.

Comparison will be performed using the Student’s t-test and chi-test for the relationship of the oxygen radical activity and severity, and Student’s t-test and logistic regression test for the relationship of the oxygen radical activity and antioxidants, and outcomes. Statistical significance will be defined as a *p*-value less than 0.05 (p<0.05).

### Inclusion criteria

Patients with severe sepsis or septic shock undergoing emergency surgery due to bowel perforation or strangulation will be screened to enroll the study. And patients requiring ICU care due to postoperative septic complications, such as pneumonia, bacteremia or peritoneal abscess or leakage. After acquesition of the informed consent, they will be assigned as to study.

### Exclusion criteria

The patients are excluded followings; (1) age < 20 years or > 80 years old; (2) other type shock except sepsis; (3) immune compromised patients, i.e., post-transplant status requiring immunosuppressant, patients using steroid due to immune disorders or other disease, patients having chemotherapeutic agents due to advanced malignancy; (4) patients who not agree the informed consent.

## Competing interests

This research is supported by Basic Science Research Program through the National Research Foundation of Korea (NRF) funded by the Ministry of Education, Science, and Technology (Grant No. 2012R1A1A2007915).

## Authors’ contributions

LJG designed and wrote the manuscript. And SH, JJY and LSH will have performed the analyses of antioxidant and oxygen radical activity, and collecting the data. All authors read and approved the final manuscript.

## References

[B1] Galley H Bench-to-bedside review: targeting antioxidants to mitochondria in sepsis Crit Care 2010 8 230 20804578 10.1186/cc9098 PMC2945094

[B2] Noveanu M Mebazaa A Mueller C Cardiovascular biomarkers in the ICU Curr Opin Crit Care 2009 8 377 383 10.1097/MCC.0b013e32832e9705 19606027

[B3] Piechota M Banach M Irzmanski R Barylski M Piechota-Urbanska M Kowalski J Plasma endothelin-1 levels in septic patients J Intensive Care Med 2007 8 232 239 10.1177/0885066607301444 17722367

[B4] Kotsovolis G Kallaras K The role of endothelium and endogenous vasoactive substances in sepsis Hippokratia 2010 8 88 93 20596262 PMC2895286

[B5] Kumar A Brar R Wang P Dee L Skorupa G Khadour F Role of nitric oxide and cGMP in human septic serum-induced depression of cardiac myocyte contractility Am J Physiol 1999 8 R265 R276 9887205 10.1152/ajpregu.1999.276.1.R265

[B6] van der Poll T van Zoelen MA Wiersinga WJ Regulation of pro-and anti-inflammatory host responses Contrib Microbiol 2011 8 125 136 21659750 10.1159/000324026

[B7] Gustot T Multiple organ failure in sepsis: prognosis and role of systemic inflammatory response Curr Opin Crit Care 2011 8 153 159 10.1097/MCC.0b013e328344b446 21346564

[B8] Galley HF Oxidative stress and mitochondrial dysfunction in sepsis Br J Anaesth 2011 8 57 64 10.1093/bja/aer093 21596843

[B9] Aksu U Demirci C Ince C The pathogenesis of acute kidney injury and the toxic triangle of oxygen, reactive oxygen species and nitri oxide Contrib Nephrol 2011 8 119 128 21921616 10.1159/000329249

[B10] Schulte J Struck J Kohrle J Muller B Circulating levels of peroxiredoxin 4 as a novel biomarker of oxidative stress in patients with sepsis Shock 2011 8 460 465 10.1097/SHK.0b013e3182115f40 21283059

[B11] Berger M Chiolero R Antioxidant supplementation in sepsis and systemic inflammatory response syndrome Crit Care Med 2007 8 S584 S590 10.1097/01.CCM.0000279189.81529.C4 17713413

[B12] Birben E Sahiner UM Sackesen C Erzurum S Kalayci O Oxidative stress and antioxidant defense World Allergy Organ J 2012 8 9 19 10.1097/WOX.0b013e3182439613 23268465 PMC3488923

[B13] Dare A Phillips A Hickey A Mittal A Loveday B Thompson N A systematic review of experimental treatments for mitochondrial dysfunction in sepsis and multiple organ dysfunction syndrome Free Radic Bio Med 2009 8 1517 1525 10.1016/j.freeradbiomed.2009.08.019 19715753

[B14] Rocha M Herance R Rovira S Hernandez-Mijares A Victor VM Mitochondrial dysfunction and antioxidant therapy in sepsis Infect Disord Drug Targets 2012 8 161 178 10.2174/187152612800100189 22420514

[B15] Hatwalne MS Free radical scavengers in anaesthesiology and critical care Indian J Anaesth 2012 8 227 233 10.4103/0019-5049.98760 22923819 PMC3425280

[B16] Crimi E Sica V Williams-Ignarro S Zhang H Slutsky AS Ignarro LJ The role of oxidative stress in adult critical care Free Radic Biol Med 2006 8 398 406 10.1016/j.freeradbiomed.2005.10.054 16443154

[B17] Abiles J de la Cruz AP Castano J Rodriquez-Elvira M Aquavo E Moreno-Torres R Oxidative stress is increased in critically ill patients according to antioxidant vitamins intake, independent of severity: a cohort study Crit Care 2006 8 R146 10.1186/cc5068 17040563 PMC1751071

[B18] Huet O Dupic L Harrois A Duranteau J Oxidative stress and endothelial dysfunction during sepsis Front Biosci 2011 8 1986 1995 10.2741/3835 21196278

[B19] Kumar Y Singh G Davidson B Free radical and antioxidant levels in patients with secondary peritonitis and their prognostic significance Dig Surg 2007 8 331 337 10.1159/000106511 17664875

[B20] Oldham KM Bowen PE Oxidative stress in critical care: is antioxidant supplementation beneficial? J Am Diet Assoc 1998 8 1001 1008 10.1016/S0002-8223(98)00230-2 9739800

[B21] Cadenas S Cadenas A Fighting the stranger-antioxidant protection against endotoxin toxicity Toxicology 2002 8 45 63 10.1016/S0300-483X(02)00381-5 12324199

[B22] Nathens A Neff M Jurkovich G Randomized, prospective trial of antioxidant supplementation in critically ill surgical patients Ann Surg 2002 8 814 822 10.1097/00000658-200212000-00014 12454520 PMC1422648

[B23] Oliveira GP Dias CM Pelosi P Rocco PR Understanding the mechanisms of glutamine action in critically ill patients An Acad Bras Cienc 2010 8 417 430 10.1590/S0001-37652010000200018 20563423

[B24] Andrews PJ Avenell A Noble DW Campbell MK Croal BL Simpson WG Randomised trial of glutamine, selenium, or both, to supplement parenteral nutrition for critically ill patients BMJ 2011 8 d1542 10.1136/bmj.d1542 21415104

[B25] Linko R Karlsson S Pettilä V Varpula T Okkonen M Lund V Serum zinc in critically ill adult patients with acute respiratory failure Acta Anaesthesiol Scand 2011 8 615 621 10.1111/j.1399-6576.2011.02425.x 21827444

[B26] Cander B Dundar ZD Gul M Girisgin S Prognostic value of serum zinc levels in critically ill patients J Crit Care 2011 8 42 46 10.1016/j.jcrc.2010.06.002 20655701

[B27] Valenta J Brodska H Drabek T Hendl J Kazda A High dose selenium substitution in sepsis: a prospective randomized clinical trial Intensive Care Med 2001 8 808 815 10.1007/s00134-011-2153-0 21347869

[B28] Giladi AM Dossett LA Fleming SB Abumrad NN Cotton BA High-dose antioxidant administration is associated with a reduction in post-injury complications in critically ill trauma patients Injury 2011 8 78 82 10.1016/j.injury.2010.01.104 20149369

[B29] Rosenfeldt F Wilson M Lee G Kure C Ou R Braun L Oxidative stress in surgery in an ageing population: pathophysiology and therapy Exp Gerontol 2013 8 45 54 10.1016/j.exger.2012.03.010 22465624

[B30] von Dessauer B Bongain J Molina V Quilodran J Castillo R Rodrigo R Oxidative stress as a novel target in pediatric sepsis management J Crit Care 2011 8 103.e1 103.e7 10.1016/j.jcrc.2010.05.001 20646907

